# The association between keloid and osteoporosis: real-world evidence

**DOI:** 10.1186/s12891-020-03898-8

**Published:** 2021-01-07

**Authors:** Chun-Ching Lu, Hao Qin, Zi-Hao Zhang, Cong-Liang Zhang, Ying-Yi Lu, Chieh-Hsin Wu

**Affiliations:** 1 Department of Orthopaedics and Traumatology, Taipei Veterans General Hospital, Taipei, 11217 Taiwan; 2 Department of Surgery, School of Medicine, National Yang-Ming University, Taipei, 11221 Taiwan; 3 Department of Neurosurgery, Zaozhuang Municipal Hospital, Zaozhuang, Shandong 277102 People’s Republic of China; 4 Department of Neurosurgery, The No.7 People’s Hospital of Hebei Province, Dingzhou, Hebei 073000 People’s Republic of China; 5 Department of Cardiology, Hebei Quyang Renji Hospital, Quyang, Hebei 073100 Taiwan; 6 Department of Dermatology, Kaohsiung Veterans General Hospital, Kaohsiung, 81362 Taiwan; 7 Shu-Zen Junior College of Medicine and Management, Kaohsiung, 82144 Taiwan; 8 Graduate Institute of Medicine, College of Medicine, Kaohsiung Medical University, Kaohsiung, 80756 Taiwan; 9 Division of Neurosurgery, Department of Surgery, Kaohsiung Medical University Hospital, Kaohsiung, 80756 Taiwan; 10 Department of Surgery, School of Medicine, College of Medicine, Kaohsiung Medical University, Kaohsiung, 80756 Taiwan

**Keywords:** Epidemiology, Inflammation, Keloid, Population-based study, Osteoporosis

## Abstract

**Background:**

Keloids are characterized by disturbance of fibroblast proliferation and apoptosis, deposition of collagen, and upregulation of dermal inflammation cells. This benign dermal fibro-proliferative scarring condition is a recognized skin inflammation disorder. Chronic inflammation is a well-known contributor to bone loss and its sequelae, osteoporosis. They both shared a similar pathogenesis through chronic inflammation. We assessed whether keloids increase osteoporosis risk through using National Health Insurance Research Database.

**Methods:**

The 42,985 enrolled patients included 8597 patients with keloids but no history of osteoporosis; 34,388 controls without keloids were identified from the general population and matched at a one-to-four ratio by age, gender. Kaplan-Meier method was applied to determine cumulative incidence of osteoporosis. Cox proportional hazard regression analysis was performed after adjustment of covariates to estimate the effect of keloids on osteoporosis risk.

**Results:**

Of the 8597 patients with keloids, 178 (2.07%) patients were diagnosed with osteoporosis while in the 34,388 controls, 587 (1.71%) were diagnosed with osteoporosis. That is, the keloids patients had 2.64-fold higher risk of osteoporosis compared to controls after adjustment for age, gender, Charlson Comorbidity Index and related comorbidities. The association between keloids and osteoporosis was strongest in patients younger than 50 years (hazard ratio = 7.06%) and in patients without comorbidities (hazard ratio = 4.98%). In the keloids patients, a high incidence of osteoporosis was also associated with advanced age, high Charlson Comorbidity Index score, hyperlipidemia, chronic liver disease, stroke, and depression.

**Conclusions:**

Osteoporosis risk was higher in patients with keloids compared to controls, especially in young subjects and subjects without comorbidities.

## Background

Keloids, which are an abnormal response to cutaneous wound healing [1], result from proliferative scars characterized by dermal fibrosis and collagen accumulation [2, 3]. Keloids often grow beyond the surrounding healthy skin and are often refractory to treatment [4]. Keloids are prone to occur in dark-skinned individuals; e.g., the estimated incidence is 4–6% in the general population but is as high as 16% in African cohorts [5]. Although its mechanisms are largely unknown, genetic susceptibility is considered the main factor in the development of this disease due to its high prevalence in certain ethnicities such as the Han-Chinese [6].

“Osteoporosis” is derived from the Greek words for “porous bone”. Osteoporosis is the most common bone disease worldwide and affects millions of people [7]. This disease is characterized by low bone mineral density (BMD) as well as deteriorated bone architecture resulting from breakup of bone homeostasis [8, 9]. Osteoporosis mostly affects middle-aged and elderly or post-menopausal women [10]. Therefore, people with osteoporosis are predisposed to bone fragility and susceptibility to osteoporotic fractures [11] resulting in substantial morbidity and mortality [12]. Additionally, osteoporosis threatens senile health; hence, osteoporosis is a worldwide public health concern.

Growing evidence shows that fibrotic diseases such as pulmonary fibrosis or scleroderma can decrease BMD. In a case-control study of a hospital database, Xie et al [13]*.*reported that pulmonary fibrosis is a risk factor for osteoporosis independent of possible confounding factors. According to recent reports, keloids share some pathobiological features with scleroderma, including inflammation and excessive collagen synthesis [14]. Di Munno et al. and La Montagna et al. reported lower than normal BMD in a study of Caucasian patients with systemic sclerosis [15, 16]. As well, Amira et al reported that patients with systemic sclerosis tended to develop osteoporosis at the distal radium and osteopenia in the lumbar area. Through chronic inflammation, impairment of bone microarchitecture and further bone mass loss develop. Hence, keloids and osteoporosis share the possible common pathophysiology by the release of inflammatory signals.

Until now, no large epidemiological studies have investigated the relationship between keloids and osteoporosis. Therefore, this study used patient data contained in the Taiwan National Health Insurance Research Database (NHIRD) to investigate whether keloid contributes to osteoporosis.

## Methods

### Data sources

Data were collected from the NHIRD Longitudinal Health Insurance Database (LHID) 2010, a subset of the NHIRD. The LHID2010 comprises data for 1 million beneficiaries randomly selected from all Taiwan National Health Insurance (NHI) beneficiaries enrolled in the NHIRD in 2010. The Taiwan NHI is a single-payer health insurance program implemented in March, 1995. It covers approximately 99% of the 23.74 million residents of Taiwan. The original medical claims in the NHIRD are available to researchers and have been used extensively for epidemiological studies in Taiwan [17]. This study of data from an encrypted secondary database was approved by the Institutional Review Board of Kaohsiung Medical University Hospital (KMUHIRB-EXEMPT (II) 20,160,016) and complied with *Declaration of Helsinki* guidelines.

All disease diagnoses were based on the International Classification of Diseases, Ninth Revision, Clinical Modification (ICD-9-CM) codes. The analysis included 8597 patients with keloids (ICD9-CM code: 701.4) diagnosed by a dermatologist or plastic surgeon for the first time [18, 19]. The occurrence of keloids was defined as > 2 diagnoses of keloids in ambulatory visits or > 1 diagnoses of keloids in inpatient care. The index date for keloids was defined as the first date of a keloids diagnosis. The analysis excluded patients aged less than 20 years old at the time of diagnosis and patients who had a history of osteoporosis (ICD-9-CM code 733) diagnosed by orthopedics with at least one BMD exam before the index date. For enhanced power in statistical analyses, particularly stratified analysis, a control group of 34,388 patients without keloids were randomly identified and matched at a ratio of 1:4 for age, gender, and index year.

### Outcome and definitions of comorbidities

All enrolled participants were followed up until the first diagnosis of osteoporosis, the end of the observation time, or the end of 2010. The occurrence of osteoporosis was defined as > 2 diagnoses of osteoporosis in ambulatory visits or > 1 diagnoses of osteoporosis in inpatient care. The impact of numerous relevant comorbidities and Charlson Comorbidity Index (CCI) scores were analyzed. A relevant comorbidity was defined as a history of diagnosis of any of the following comorbidities in the claims records data before the index date: hyperlipidemia (ICD-9-CM code 272), hypertension (ICD-9-CM codes 401–405), diabetes mellitus (ICD-9-CM code 250), chronic liver disease (ICD-9-CM codes 571.2, 571.4–571.6, 456.0–456.21, 572.2–572.8), chronic kidney disease (ICD-9-CM codes 582,583,585,586 and 588), chronic pulmonary disease (ICD-9-CM codes 490–496), hyperthyroidism (ICD-9-CM code 242), hyperparathyroidism (ICD-9-CM code 252), stroke (ICD-9-CM codes 430–438), depression (ICD-9-CM codes 296.2, 296.3, 300.4 and 311;), alcohol attributed diseases (ICD-9-CM codes 291.0–9, 303, 305.0, 357.5, 425.5, 535.3, 571.0–3, 980.0 and V11.3), obesity (ICD-9-CM code 278), tobacco use disorder (ICD-9-CM code 350.1). Based on the CCI scores, the severity of comorbidities was classified as 0, 1, 2 or > 3, where 0 and > 3 were defined as the lowest and highest severity of comorbidity, respectively.

### Statically analysis

First demographic data for the enrolled population were analyzed. Student t test and Wilcoxon rank-sum test were utilized to estimate continuous variables, including mean age and follow-up time (y) while Chi-square test was utilized to examine categorical variables in clinical characteristics between the two cohorts. Kaplan-Meier method was employed to measure cumulative incidence of osteoporosis, and 2-tailed log rank test was utilized to analyze between-group differences. In keloids patients, the survival period was calculated from the index date of keloids until the occurrence of an ambulatory visit or hospitalization for osteoporosis, or the end of the study period (December 31, 2010), whichever came first. Incidence rates of osteoporosis were expressed in 1000 person-years and compared by Poisson regression analyses. Cox proportional hazard regression models were applied to derive the hazard ratio (HR) with corresponding 95% confidence interval (CI) for the association between keloids and the risk of developing osteoporosis after adjusting covariates including age, gender, CCI score, and relevant comorbidities (hyperlipidemia, hypertension, diabetes mellitus, chronic liver disease, chronic kidney disease, chronic pulmonary disease, hyperthyroidism, hyperparathyroidism, stroke, depression, alcohol attributed diseases, obesity, and tobacco use disorder). All analyses in the study were conducted using SAS version 9.4 (SAS Institute, Cary, NC, USA). A two-tailed *p*-value less than 0.05 was considered statistically significant.

## Results

### Baseline characteristics

Table 1 compares the baseline characteristics of the keloids group and the control group. In total, 8597 keloids patients and 34,388 controls without keloids (mean age 34.6 + 13.5 and 34.7+ 13.7 years, respectively) were enrolled for analysis. Females comprised 62.37% of all participants and approximately half (50.04%) were less than 30 years old. Compared to the control group, the keloids group had a higher CCI score and significantly more patients with related comorbidities.

**Table 1:** Baseline characteristics of the keloids group and the control group

Variables	Keloid		*P* value
	Yes	No	
	*N*=8597	*N*=34,388	
Osteoporosis patients, n (%)	178 (2.07)	587 (1.71)	< 0.05
Period of developing osteoporosis median (IQR^a^), years	3.0 (1.2–5.7)	6.7 (4.1–10.2)	< 0.001
Mean age of osteoporosis (SD^b^), years	56.3 (14.0)	65.5 (14.8)	< 0.001
Age mean (SD^b^), years	34.6 (13.5)	34.7 (13.7)	0.299
Age group, n (%)			
20–29	4302 (50.04)	17,208 (50.04)	
30–39	1939 (22.55)	7756 (22.55)	
40–49	1194 (13.89)	4776 (13.89)	
50–59	617 (7.18)	2468 (7.18)	
60–69	309 (3.59)	1236 (3.59)	
> 70	236 (2.75)	944 (2.75)	1.000
Sex, n (%)			
Males	3235 (37.63)	12,940 (37.63)	
Females	5362 (62.37)	21,448 (62.37)	1.000
Charlson Comorbidity Index, n (%)			
0	2494 (29.01)	16,286 (47.36)	
1	3963 (46.10)	13,710 (39.87)	
2	1389 (16.16)	3018 (8.78)	
≥3	751 (8.74)	1374 (4.00)	< 0.001
Co-morbidity, n (%)			
Hyperlipidemia	1771 (20.60)	4197 (12.20)	< 0.001
Hypertension	1513 (17.60)	3818 (11.10)	< 0.001
Diabetes mellitus	1049 (12.20)	2533 (7.37)	< 0.001
Chronic liver disease	2186 (25.43)	4947 (14.39)	< 0.001
Chronic kidney disease	568 (6.61)	1352 (3.93)	< 0.001
Chronic pulmonary disease	2887 (33.58)	8370 (24.34)	< 0.001
Hyperthyroidism	643 (7.48)	1424 (4.14)	< 0.001
Hyperparathyroidism	19 (0.22)	35 (0.10)	0.005
Stroke	262 (3.05)	530 (1.54)	< 0.001
Depression	1066 (12.40)	2274 (6.61)	< 0.001
Alcohol attributed disease	232 (2.70)	632 (1.84)	< 0.001
Obesity	227 (2.64)	591 (1.72)	< 0.001
Tobacco use disorder	65 (0.76)	122 (0.35)	< 0.001

^a^ *IQR* Interquartile range; ^b^ *SD* Standard deviation;

Furthermore, the keloids group had a significantly (*P*< 0.05) higher incidence of osteoporosis compared to the control group. Out of 8597 patients in the keloids group, 178 (2.07%) had osteoporosis. In contrast, out of the 34,388 patients in the control group, 587(1.71%) had osteoporosis. Osteoporosis developed significantly faster in the keloids group (3.0 years) compared to the control group (6.7 years) during subsequent periods. Osteoporosis were diagnosed at a significantly younger age in the keloids group compared to the control group (56.3 vs. 65.5 years, respectively; *P*< 0.001).

**Fig. 1:**
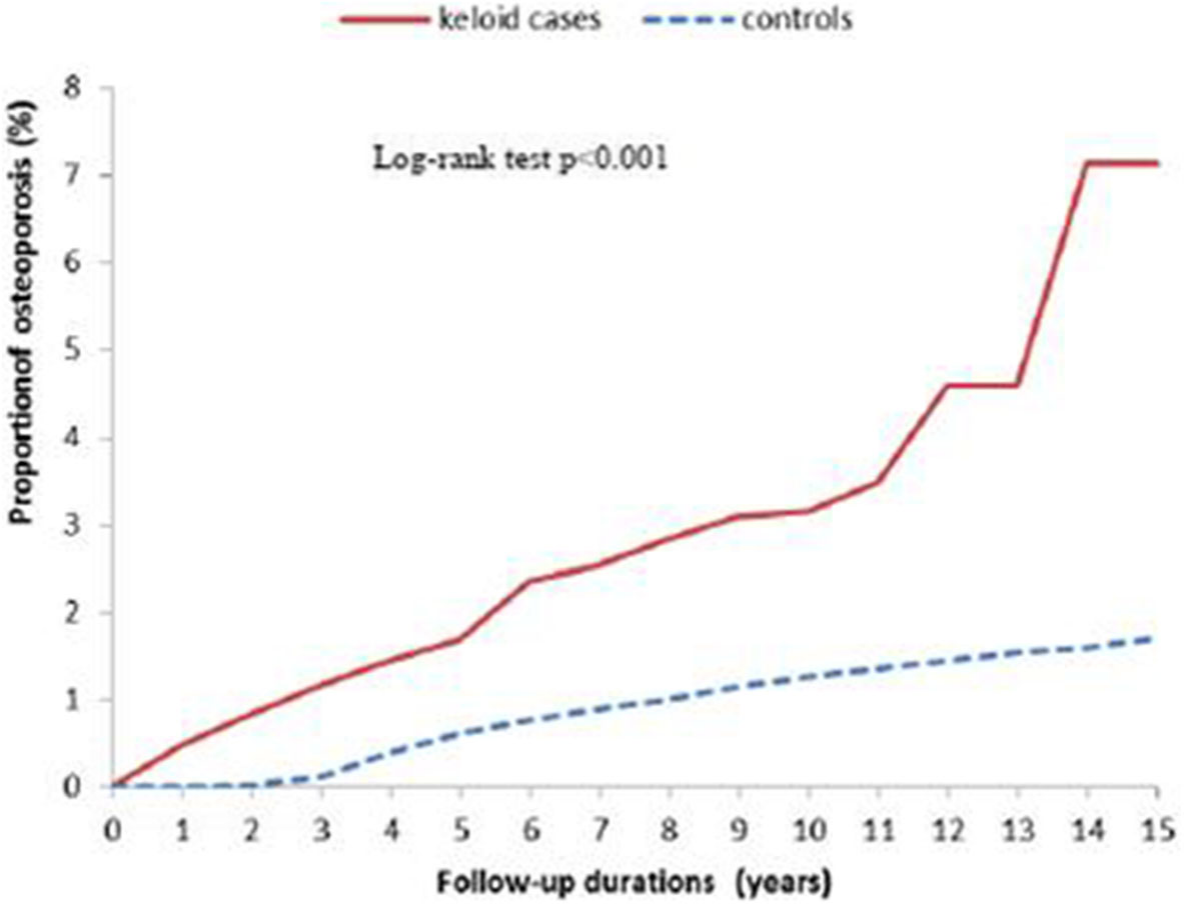
Cumulative incidence of osteoporosis among patients with keloids and the control cohort

Figure 1 presents the 15-year probability for osteoporosis associated with keloids. Kaplan–Meier survival analysis with a log rank test revealed that keloids was significantly associated with subsequent osteoporosis (*P* < 0.001).

Table 2 stratifies the osteoporosis incidence and risk in keloids patients by age, gender and comorbidities.

**Table 2:** Keloids associated with osteoporosis stratified by age, gender and comorbidities

Variables	Patients with keloid			Patients without keloid			Compared with non-keloid	
	Osteoporosis	PYs	Rate	Osteoporosis	PYs	Rate	IRR (95% CI)	Adjusted HR^a^ (95% CI)
All	178	49,037.42	3.63	587	511,351.32	1.48	3.16 (2.67–3.74)^*^	2.64 (2.21–3.17)^*^
Gender								
Men	43	18,664.24	2.30	144	193,172.89	0.75	3.09 (2.19–4.34)^*^	2.97 (2.04–4.33)^*^
Women	135	30,373.18	4.44	443	318,178.43	1.39	3.19 (2.63–3.87)^*^	2.56 (2.08–3.15)^*^
Age								
20–49	61	43,388.67	1.41	64	445,735.38	0.14	9.79 (6.89–13.90)^*^	7.06 (4.93–10.11)^*^
> 50	117	5648.75	20.71	523	65,615.94	7.97	2.59 (2.13–3.18)^*^	2.06 (1.67–2.54)^*^
Comorbidity								
No	9	16,842.54	0.53	40	279,314.00	0.14	3.73 (1.81–7.69)^*^	4.98 (2.40–10.29)^*^
Yes	169	32,194.88	5.25	547	232,037.32	2.36	2.23 (1.87–2.65)^*^	2.54 (2.11–3.06)^*^

*PYs* Person-year; Rate, incidence rate in per 1000 person-years; *IRR* Incidence rate ratio in per 1000 person-years; *95% CI* 95% Confidence interval; *HR* Hazard ratio

^a^ Model adjusted for age, gender, Charlson Comorbidity Index and relevant comorbidities

* *P*< 0.001

In the keloids group, 178 individuals had osteoporosis, which was an incidence density of 3.63 per 1000 person-years. In the control group, 587 individuals had osteoporosis, which was an incidence density of 1.48 per 1000 person-years. The keloids group had a 2.64-fold greater risk of osteoporosis compared to controls after adjustment for age, gender, CCI score, and related comorbidities.

Gender-specific analyses showed that the incidence of osteoporosis was higher in women than in men in both the keloids group (4.44 vs. 2.30 per 1000 person-years, respectively) and the control group (1.39 vs. 0.75 per 1000 person-years, respectively). Furthermore, osteoporosis risk was significantly higher in the keloids group compared to the control group in both women (adjusted HR=2.56, 95% CI: 2.08–3.15, *P*< 0.001) and men (adjusted HR=2.97, 95% CI: 2.04–4.33, *P*< 0.001).

Age-stratified analyses revealed that the incidence of osteoporosis consistently increased with age in both cohorts. Patients younger than 50 years (adjusted HR=7.06, 95% CI=4.93–10.11, *P*< 0.001) and the elderly aged 50 years and older (adjusted HR=2.06, 95% CI=1.67–2.54,

*P*< 0.001) were prone to developing osteoporosis. Nevertheless, younger patients were at significantly higher risk than were older patients.

The comorbidity-stratified analyses revealed that the keloids group had a higher risk of osteoporosis compared to the control group. The osteoporosis risk contributed by keloids was decreased in the presence of comorbidity.

Table 3 depicts the risk of developing osteoporosis in keloids patients.

**Table 3:** Significant predictors of osteoporosis after keloid

Variables	Adjusted HR ^a^	(95% CI)	*P*
Age (in 10-year interval)	2.07	(1.85–2.33)	< 0.001
Female gender	4.17	(2.91–5.98)	< 0.001
High Charlson Comorbidity Index	1.53	(1.23–1.89)	< 0.001
Hyperlipidemia	1.79	(1.27–2.53)	< 0.001
Stroke	1.79	(1.21–2.66)	< 0.01
Chronic liver disease	1.54	(1.12–2.11)	< 0.01
Depression	1.57	(1.12–2.19)	< 0.01

*HR* Relative hazard ratio; *95% CI* 95% Confidence interval

^a^ Model adjusted for age, gender, Charlson Comorbidity Index and relevant comorbidities

A Cox regression model was used to identify potential risk factors for osteoporosis in keloids patients. Table 3 shows that predictive factors included older age, female gender, high CCI scores, hyperlipidemia, stroke, chronic liver disease, and depression.

The effects of comorbidities on the association between keloids and osteoporosis risk were further explored by adjusting these covariates and stratifying risk by these comorbidities. A comparison of the keloids group and the control group revealed that risk factors for osteoporosis were hyperlipidemia, chronic liver disease, stroke and depression (Table 4).

**Table 4:** The osteoporosis incidence and risk in keloids patients with comorbidity

Variables	Patients with keloids			Patients without keloids			Compared with non-keloids controls
	Osteoporosis	PY	Rate	Osteoporosis	PY	Rate	Adjusted HR(95% CI)^a^
**Hyperlipidemia**							
No	60	38,378.15	1.56	251	451,138.90	0.57	2.48 (1.85–3.33)^*^
Yes	118	10,659.27	11.07	336	60,212.42	5.58	2.74 (2.20–3.42)^*^
**Stroke**							
No	143	47,551.35	3.01	501	504,095.08	0.99	2.59 (2.13–3.16)^*^
Yes	35	1486.07	23.55	86	7256.23	11.85	2.90 (1.95–4.33)^*^
**Chronic liver disease**							
No	81	35,472.65	2.28	338	439,113.30	0.77	2.34 (1.82–3.01)^*^
Yes	97	13,564.77	7.15	249	72,238.01	3.45	2.99 (2.34–3.81)^*^
**Depression**							
No	125	42,773.81	2.92	467	478,243.51	0.98	2.45 (1.98–3.02)^*^
Yes	53	6263.61	8.46	120	33,107.81	3.62	3.30 (2.37–4.61)^*^

*PYs* Person-year; Rate, incidence rate in per 1000 person-years; *95% CI* 95% Confidence interval; *HR* Hazard ratio

^a^ Model adjusted for age, gender, Charlson Comorbidity Index and relevant comorbidities

^*^ *P*< 0.001

Table 5 shows the effect of keloids and comorbidities on the risk of osteoporosis development. The analyses suggested that keloids and comorbidities jointly affected the subsequent development of osteoporosis.

**Table 5:** Joint effect of keloid and comorbidities on the risk of osteoporosis

Variables		N	Osteoporosis	Rate	Adjusted HR (95% CI)
**Keloid**	**Hyperlipidemia**				
No	No	30,191	251	0.56	1.00 (Reference)
No	Yes	4197	336	5.58	1.37 (1.13–1.65)^**^
Yes	No	6826	60	1.56	2.48 (1.85–3.33)^**^
Yes	Yes	1771	118	11.07	3.75 (2.93–4.80)^**^
**Keloid**	**Chronic liver disease**				
No	No	29,441	338	0.77	1.00 (Reference)
No	Yes	4947	249	3.45	1.19 (1.00–1.43)^#^
Yes	No	6411	81	2.28	2.34 (1.82–3.01)^**^
Yes	Yes	2186	97	7.15	3.56 (2.77–4.58)^**^
**Keloid**	**Stroke**				
No	No	33,858	501	0.99	1.00 (Reference)
No	Yes	530	86	11.85	1.45 (1.14–1.85)^*^
Yes	No	8335	143	3.01	2.59 (2.13–3.16)^**^
Yes	Yes	262	35	23.55	4.21 (2.94–6.03)^**^
**Keloid**	**Depression**				
No	No	32,114	467	0.98	1.00 (Reference)
No	Yes	2274	120	3.62	1.18 (0.96–1.46)^#^
Yes	No	7531	125	2.92	2.45 (1.98–3.02)^**^
Yes	Yes	1066	53	8.46	3.92 (2.90–5.29)^**^

^a^ Rate, incidence rate in per 1000 person-years; *95% CI* 95% Confidence interval; *HR* Hazard ratio

^*^ *P*< 0.01, ^**^P< 0.001, ^#^ non-significant

## Discussion

The results of this study indicated that keloids were associated with an increased sequential risk of osteoporosis. To the best of our belief, this is the first study designed to examine the association between keloids and osteoporosis in an Asian population. Compared to the control group, the keloids group had a 2.64-fold higher osteoporosis risk after adjusting for covariates. As expected, the incidence of subsequent osteoporosis in keloids patients was higher in females than in males (4.44 vs. 2.30 per 1000 person-years, respectively). The association between keloids and osteoporosis was much stronger in patients younger than 50 years compared to those older than 50 years (HR= 7.06%) and was much stronger in patients without comorbidities compared to those with comorbidities (HR = 4.98%). In keloids patients, old age, female gender, high CCI score, hyperlipidemia, chronic liver disease, stroke and depression were associated with an increased incidence of osteoporosis.

Several possible mechanisms could underlie the increased osteoporosis risk observed in the keloids group. First, 1,25-dihydroxyvitamin D (1,25 (OH)_2_ D_3_) is the active metabolite of vitamin D, which is critical in cell proliferation and differentiation, collagen synthesis and degradation [20], hormone secretion, calcium homeostasis as well as bone remodeling [21]. Recent studies show an association between vitamin D and keloids [22]. Zhang et al [23]*.*reported that vitamin D and its metabolites can decrease fibrosis in keloids through vitamin D receptors (VDR) while 1,25 (OH)_2_ D_3_ inhibits extracellular matrix deposition and matrix-metalloproteinase activity induced by transforming growth factor (TGF)-β. In 2013, Yu et al [24]*.*reported the presence of TagI gene polymorphisms of VDR and lower levels of serum circulating 1,25 (OH)_2_ D_3_ in Chinese patients with keloids. Carriers of the TagI CC genotype had a higher keloids risk and significantly lower serum 1,25 (OH)_2_ D_3_ compared to carriers of other genotypes. Gong et al [25]*.* also reported that high levels of plasminogen activator inhibitor-1 and low levels of VDR expressions were significantly associated with keloids development. Since vitamin D is important for maintaining bone health, vitamin D insufficiency and hyperparathyroidism are the main causes of osteoporosis in men and pre-menopausal women [26]. Low vitamin D levels are independently associated with a 7.5-fold increase in osteoporosis risk [27]. Patients who have prolonged vitamin D deficiency are predisposed to osteoporosis. Therefore, decreased serum vitamin D level may also contribute to osteoporosis risk in keloids patients.

Second, chronic inflammation caused by keloids inhibits osteoblast growth, which can lead to osteoporosis [28]. Arima et al. indicated that hypertension is associated with severity of keloids. In a study of 340 keloids patients, ordinal logistic regression analyses revealed that blood pressure correlated positively with both the number and size of keloids. The results implied that hypertension may aggravate keloids by increasing inflammatory responses in tissues while hypertension damages blood vessels [29, 30]. Moreover, histology studies show that keloids illustrates proliferation of fibroblasts, excess production of collagen, formation of new vessels, and inflammation of dermal cells, which reveals continuous inflammation in abnormal wound healing processes [31, 32]. In comparison with normal skin, keloids have a larger number of T cells, a higher CD4/CD8 ratio [14], and more persistent peripheral blood lymphocytes, which accelerate collagen production by dermal fibroblasts [33]. The release of proinflammatory cytokines (interleukin (IL)-1, IL-6 and tumor necrosis factor (TNF)-α) in keloid tissues increases sensitivity to injury and upregulates these cytokines compared to the general population [34, 35] . Redlich et al [36]*.* reported that chronic inflammation in the body could influence bone tissue metabolism and eventually cause bone loss. These cytokines inhibit bone collagen synthesis and are potent stimulators of osteoclast-induced bone resorption, which are central in the context of chronic inflammation [37]. Accordingly, keloids increase osteoporosis risk via cytokines produced by chronic inflammation.

Third, micro-RNA (miRNA), a class of non-coding small RNA, can control gene expression by binding target mRNAs and are crucial in cellular hemostasis [38, 39]. Aberrant miRNA expression reportedly contributes to both the development of keloids and the pathogenesis of osteoporosis [40–46]. Additionally, miR-21 has a central role in wound healing [47]. After tissue injury, miR-21 expression is induced by TGF-β1 [48]. The miR-21 is up-regulated in keloid tissues and can regulate keloidal fibroblast proliferation and apoptosis [49–51]. In 2014, Seeliger et al. identified significantly higher than normal expressions of miR-21 in serum and bone tissues in patients with osteoporosis. Additionally, miR-21 reportedly promotes osteoclastogenesis by participating in the RANKL-induced differentiation of osteoclasts [52]. Sugatani et al [53]*.* also reported that miR-21 has a crucial role in estrogen-controlled osteoclastogenesis. Consequently, micro-RNA has essential contributing roles in the occurrence of keloids and osteoporosis.

Finally, several reports indicate that major depression and depressive symptoms adversely affect bone density, which increases bone fracture risk. The relationship between depression and BMD has also been illustrated in elderly Caucasian women and in Asian men [54, 55]. In recent Taiwan studies, Lee et al. reported that depression patients had a 1.30-fold greater chance of developing osteoporosis compared to controls without depression, and Huang et al. reported that patients with post-traumatic stress disorder had a 2.66-fold higher likelihood of developing osteoporosis compared to controls without this disorder [56]. Previous studies suggest that depression can cause osteoporosis through dysregulation of the hypothalamic-pituitary-adrenocortical axis, parathyroid hormones and cytokines. In Furtado et al. [57], the Hospital Depression and Anxiety Scale administered before and after surgery revealed that psychological stress influenced recurrence of keloids. Therefore, depression is a significant risk factor for osteoporosis in keloids patients, which is consistent with the results of our analyses.

There are several clinical implications for human health in our study. First, physicians might assess keloids patients for osteoporosis from diagnosis to follow-up due to the increased risk. Second, measuring BMD is an easily-performed noninvasive technique and gives accessory glues to assess future fracture risk, which can remind physicians to early recognition of osteoporosis. As keloids increased the risk of osteoporosis, particularly in keloids and comorbidities (hyperlipidemia, chronic liver disease, stroke and depression), physician may arrange the osteoporosis survey for those susceptible patients with complaints to improve outcome and quality of life.

A notable strength of this study is the use of a large-population database, which provided sufficient statistical power. Moreover, use of administrative data eliminated the potential for volunteer or selection bias. Finally, since the majority of patients in the two cohorts were ethnic Chinese, in which human leukocyte antigen polymorphism has a strong impact on keloid susceptibility with different ethnics, further analyses of data for this population may reveal genetic factors in the development of keloids and their role in osteoporosis. However, several limitations of this study should be addressed. First, the database lacked personal information such as tobacco use, dietary supplements, calcium intake, body mass index, physical activity, socioeconomic status, and laboratory data, which could have biased the analyses. Second, the severity (size or number), location and the nature of keloids were not disclosed in the database. Hence, this study could not determine how these factors affect the occurrence of osteoporosis. A further prospective study is needed to validate this association. Third, the information of detailed medication was incomplete in the NHIRD database. The association of keloids and osteoporosis might be confounded by treatment of keloid unless patients receive large cumulative dosage of corticosteroids. However, the total amount of corticosteroids is hard to identified and is usually too small to induce osteoporosis. Therefore, the weight of the confounder by medication to keloids could be diluted by other factors causing by keloids itself.

## Conclusions

This study is the first to analyze the association between keloids and osteoporosis risk in an Asian population. The analyses showed that keloids patients had a 2.64-fold higher osteoporosis risk compared to controls, suggesting keloids, a common disorder in Asian, would be an early predictor of osteoporosis. Additionally, among patients with younger age (less than 50 years) and those without comorbidities, the probability of osteoporosis was much higher than in the counterpart. In the keloids group, risk factors for osteoporosis included old age, female gender, high CCI score, hyperlipidemia, chronic liver disease, stroke and depression. Hence, counseling and management are suggested for helping keloids patients improve osteoporosis symptoms (e.g., backache and joint pain) and for preventing known complications (e.g., fractures).

## Data Availability

The datasets generated and/or analysed during the current study are not publicly available due to the Taiwan Personal Information Protection Act, but are available from the corresponding author on reasonable request. All relevant data to support the study findings are provided in this article.

## Abbreviations

BMD: Bone mineral density
CCI: Charlson comorbidity index
ICD-9-CM: International classification of diseases, ninth revision, clinical modification
IL: Interleukin
LHID: Longitudinal health insurance database
miRNA: Micro-RNA
NHIRD: National health insurance research database
TGF: Transforming growth factor
TNF: Tumor necrosis factor
VDR: Vitamin D receptors
